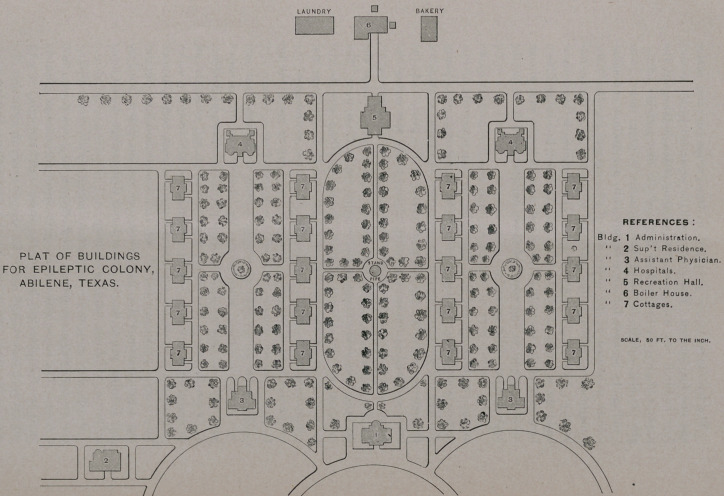# The Founding of a State Institution for Epileptics in Texas*A paper read at the first annual meeting of the National Association for the Study of Epilepsy and the Care and Treatment of Epileptics, held in Washington, D. C., May 14 and 15, 1901.

**Published:** 1902-09

**Authors:** B. M. Worsham

**Affiliations:** Superintendent of the State Lunatic Asylum at Austin


### TEXAS MEDICAL JOURNAL.

ESTABLISHED JULY, 1885.

PUBLISHED MONTHLY.—SUBSCRIPTION $1.00 A YEAR.

VOL. XVIII. AUSTIN, SEPTEMBER, 1902. No. 3.

### Original Contributions.

For Texas Medical Journal.

### The Founding of a State Institution for Epileptics in Texas.*

BY B. M. WORSHAM, M. D.,
Superintendent of the State Lunatic Asylum at Austin.

In preparing a paper to be read before this Association, I find myself confronted with many difficulties. Not having had experience in the management of an institution specially designed for the exclusive care and treatment of epileptics, and my experience in the treatment of epilepsy having been largely confined to a limited number of cases of the insane class, it is impossible for me to offer any suggestions, either as to the proper management of an institution or the treatment of this disease. For the past two or three years, however, I have devoted considerable time and study to the arrangement of grounds, buildings, and other equipments necessary in carrying out the advanced ideas of the colony plan adopted and so successfully operated in New York and other States; therefore I shall speak only of what has been done in Texas toward the establishment of an epileptic institution—the plans adopted for the various buildings, their arrangement and location, together with some reasons for such arrangement.

Since the establishment of Craig Colony, the advisability of creating a similar institution in Texas has been more or less discussed by the medical profession and others in our State. Two

*A paper read at the first annual meeting of the National Association for the Study of Epilepsy and the Care and Treatment of Epileptics, held in Washington, D. C., May 14 and 15, 1901.

years ago a bill was passed by the Legislature making an appropriation of $100,000 for an epileptic colony, and accepting an offer made by the citizens of the town of Abilene, Taylor county, of 640 acres of land upon which the proposed institution was to be located. The bill provided that a committee should be selected by the Governor to select the site. The committee was appointed, and the location made three miles from the limits of the city of Abilene. The bill had an objectionable feature, in that it stipulated that the amount of $100,000 should provide accommodation for five hundred patients. On this account, after plans had been prepared, no attempt was made to begin the construction of the buildings, as it was against the policy of the Governor to erect cheap and defective structures.

At a special session of the Legislature, called to meet some eight or ten months later, the limit to the cost of the institution was removed; but the demand that five hundred patients shall be provided for was left in the bill. Plans were perfected and bids advertised for, the lowest bid being nearly $500,000. This amount was so far in excess of what it was thought the Legislature had in mind the institution would cost, that all bids were rejected, and active operations were deferred, in order to allow the Legislature to pass upon the advisability of so large a sum of money being expended at one time for this purpose. Finally the bill was amended by striking out the number of patients to be provided for and appropriating $200,000. Bids will again be advertised for at an early date, and as many of the buildings completed as the appropriation of $200,000 will permit.

A complete plot of the grounds and the proposed buildings has been made and adopted, showing the location of each building, the recreation park with drives and walks, the farm, garden, etc.; and this plot will be used as a guide for the future development of the institution.

The location possesses many advantages. The land is suited to all kinds of farming and gardening, and is especially adapted to raising all kinds of fruit. It will be possible to procure more land adjoining the tract, and I have advised that a much larger tract be purchased before the institution is opened for the admission of patients. The site selected for the buildings is an ideal one for good drainage; and the scenery and climate are unsurpassed in Texas, if not in the South.

I have had prepared a plan of the colony and I herewith present it for your consideration. I think it will give you a better idea of the arrangements than any verbal description could do.

PLAT OF BUILDINGS

FOR EPILEPTIC COLONY,

ABILENE, TEXAS.

**REFERENCES:**

Bldg. **1** Administration.

" **2** Sup't Residence.

" **3** Assistant Physician.

" **4** Hospitals.

" **5** Recreation Hall.

" **6** Boiler House.

" **7** Cottages.

The buildings will be arranged in two groups—one for males and one for females. The groups will be separated by an avenue four hundred feet wide, with the administration building at the upper end, seventy-five feet in front of the first cottages of the groups, and equally dividing the distance between them. The building to be used for an assembly hall, gymnasium and natatorium is located at the lower end of this avenue in exactly the same relative position to the lower end of the groups as the administration building is to the front. The distance between these buildings is six hundred and fifty feet, the grade line being fifteen feet lower at the assembly hall and rising to its height with almost uniform grade to the administration building, which is on the highest point of the grounds.

There are two rows of cottages in each group, separated by an avenue two hundred and fifty feet wide. At the upper end of this avenue and twenty-five feet to the front of the cottages, equally dividing the distance between the two rows, is located a residence for assistant physician and officers in charge of this group. At the lower end, and in the same position relative to the rear end as the building just described is to the front end, is the infirmary building for this group. Each row consists of five cottages, and the distance from one cottage to the next is one hundred feet. The residence of the superintendent is located in front and to the extreme right of the colony, about six hundred feet from the administration building. In the rear of all the buildings of the colony are located the power, heating, and lighting plant, the ice factory and cold storage building, the laundry, etc.

The water supply will be obtained from an artifical lake one mile from the colony, which covers six hundred and fifty acres of land, and is the source of the water supply for the town of Abilene.

To recapitulate: The colony when completed will consist of an administration building, twenty cottages, one infirmary for males and one for females, the superintendent's residence, two residences for assistant physicians and officers, an assembly building containing a gymnasium and a natatorium, one central power, heating, and lighting plant, the ice factory and cold storage department, industrial buildings, barns, and dairy.

A complete system of sewerage connecting with each building merges into the main sewer, which extends to a creek one mile distant. The advisability of laying the main sewer on an embankment two feet above the level of the ground, and arranging it with gates two hundred feet apart for the purpose of irrigating the gardens on either side, is now under consideration. A plan similar to this, but on a smaller scale, is in operation at the Southwestern Hospital for the Insane, located at San Antonio, Texas, and the results have been satisfactory.

The buildings are designed to have plain brick walls, hipped roofs, with broad, overhanging cornices heavily bracketed. The basement-to-sill course will be faced with Pecos red sandstone. The walls will be of light-buff brick and the roofs of gray slate.

The entrance to the administration building will be through an ornamental porch, the decorations of which will be Texas flowers and emblems conventionalized. The residences will be more ornamental, and of a style best adapted to the heat of the country, with galleries extending almost entirely around the buildings, and with projecting cornices. All the buildings are to have fire-proof construction of floors, ceilings, and roofs.

All halls and rooms in the cottages will be finished with quarter-sawed pine floors, enamel brick wainscoting five feet high, and walls plastered with Keene cement. The ceilings and cornices will be constructed of ornamental steel panels; all jambs to windows and doors will be rounded, and no woodwork except floors, sash and doors, and the least possible framing will appear. The stairs will be of open iron construction, with slate treads and landings and wood-finished handrails. Toilet-rooms will have floors of vitreous tile, and walls finished with enamel brick. All plumbing is to be of the best quality and placed in the most skillful manner.

Special attention will be given to the interior finish of the infirmary buildings, with a view to the best sanitary conditions. The plastering will be troweled down to a smooth marble-like surface and afterwards painted with three coats of the best enamel paint.

The cottages are to be two stories in height with basements six feet above the ground and four feet below. In each basement story we propose to locate the kitchen, dining rooms, storeroom, pantries, etc. The first story will consist of a large hall, a large dayroom, two single rooms, one room that will accommodate about four patients, and the toilet rooms. The second floor will have two dormitories with room for ten or twelve beds each, two single rooms, a wide hall, clothing and linen rooms, a bathroom and closet.

After careful investigation and study of the needs of an institution of this character in the way of machinery and apparatus for motive power, heating, ventilation, refrigeration and water supply, I have decided that the most practical plan to adopt is to build a centrally located power house, as shown on the plot plan of the buildings and grounds. The location of the power house is such that the steam and water lines, the electric wires, etc., running to the various buildings will not be any longer than is necessary, and that all pipe lines will drain toward the power house. In this building will be placed the boilers, engines, dynamos, pumps, water heater, etc.

The pipes for conveying steam to the heating and ventilating apparatus in the various buildings, together with the hot and cold water pipes, and the electric wires, will be run in tunnels leading from the power house to the other buildings. The tunnels will be of ample size, so that the pipes and wires can be installed after the tunnels are completed, and so that they can be inspected readily and repairs made at any time.

The boilers will be of the safety-water-tube type, divided into four units of two hundred horse-power each, and will be equipped with fuel-saving, smokeless furnaces. It is the intention to keep one unit in reserve at all times. These boilers will furnish steam to operate the light engines, pumps, heating and cooking apparatus.

There will be three electric light engines and dynamos of the direct-connected type. One of these units will carry the minimum load by itself and two of them running together will carry the maximum load, leaving one unit in reserve at all times, in order to prevent a shut-down in case of repairs. These machines will furnish electric current, not alone for lighting the buildings and grounds, but also current to operate the electric motors to run the ice plant, the water pumps, the laundry machinery, the fans, and the heating and ventilating apparatus in the various buildings, and any other motors that may be required for operating machinery in the industrial buildings. It was decided to have electric transmission of power on account of economy in operating widelv scattered machinery in preference to any other method that could be employed. The control of the various circuits for lighting as well as for motive power will be such that current can be turned on or off at any machine or in any section of a building without interfering with the operation of any of the other machines or apparatus of any building or section of a building.

Two pumps driven by electricity will be placed at the lake and connected with the power house by wires. Each of these pumps will be of ample capacity to supply the maximum amount of water required daily, keeping one in reserve at all times in case repairs have to be made to the other. The water will be pumped to a standpipe, as shown on the plot plan, and from the standpipe will be distributed through a system of pipes to the various buildings and grounds.

A fire pump will be placed in the power house, the suction of the same being connected with the stand pipe; and a system of waterpipes will be distributed throughout the grounds with fire plugs placed near the various buildings. This pump will be automatically controlled, so that when the water is turned on at any of the fire plugs the pump will be automatically started, thus maintaining the desired pressure for fire protection at all times.

The various buildings will be heated and ventilated by means of the fan system, the fans being operated by electric motors. Fresh air will be introduced from the outside of each building through screens, then passed over heaters, which consist of large coils of one-inch steam pipe, and after being warmed to the desired temperature the air will be forced into the warm air flues built in the walls of the rooms, and delivered through registers in the various rooms. Ventilating flues will lead from each room to the attic, where they will be connected with a main horizontal duct, which, in turn, connects with a large ventilator in the roof of the building. Each heating apparatus is arranged with a by-pass connection, so that fresh, tempered air or hot air can be delivered into the various rooms as the case may require, depending on the temperature to be maintained. The admission of hot and tempered air is to be controlled automatically by means of the Johnson system of temperature regulation, and the apparatus so arranged that any desired temperature can be maintained in any apartment in any building, regardless of the outside temperature, and so that there will be a continuous circulation, the air supply being sufficient to secure a complete change of air in the building every ten minutes. The warm air is delivered at a point about eight feet from the floor, and the foul air is taken out close to the floor line through wrought iron screens or registers. No radiators are placed in any of the rooms or corridors.

The exhaust steam from the engines and pumps will be utilized in the heating apparatus to the fullest extent by means of the American automatic vacuum system, and when the supply of the exhaust steam is insufficient, live steam will be admitted automatically, to make up the deficiency. By this system exhaust steam is utilized without back pressure on the engines, being circulated throughout the entire apparatus at atmospheric pressure. In this way a great saving is made in fuel, and a higher efficiency is secured in the circulation of steam throughout the system than could be secured by the ordinary methods of heating.

The water of condensation from the various buildings and apparatus is drawn back to the boiler room by means of a vacuum pump, to which the main return pipes are connected. This pump delivers the condensation into the feed water heater, from which it is pumped back into the boilers. In this way not alone is a saving in fuel accomplished, but there is a large saving in water; and by returning distilled water to the boilers, scale is practically prevented. This system is in use in the State Lunatic Asylum at Austin, Texas, and in several of the other State institutions, where it is giving excellent results.

The ice plant will be of the latest improved design and will be of ample capacity to furnish ice for the institution, as well as for the refrigeration of the cold storage rooms, for meats, vegetables, etc.

In preparing the plans and locating the buildings in only two groups, as described, I am free to confess that I was prompted largely by economy in construction and subsequent operations. I was aware that the original colony idea carried with it the locating of cottages, or groups of cottages, at random and widely separated; but an institution arranged in that way would undoubtedly cost a great deal more to operate than would one arranged after the plan we have adopted. The former plan precludes the possibility of successfully operating a central heating plant, by which all exhaust steam that would otherwise be lost can be utilized for heating purposes, as can be done by the low pressure vacuum system that we propose to install.

Aside from an economic point of view, it seems to me that, with an institution scattered over two thousand or three thousand acres of land, the superintendent or his assistant physicians could not give the necessary personal supervision to each department of the colony that would insure their familiarity with the care and attention the patients were receiving at the hands of employes. By having only two groups of cottages, with a distance of one hundred feet from one cottage to the next, not only are beauty and symmetry secured to the institution, but it is possible to carry out the essential ideas that I have heard advanced in favor of the colony plan, namely: placing in each building properly classified patients, not to exceed twenty-five or thirty in number, and having the affairs of each cottage conducted as a separate and distinct household, with its kitchen, dining room, etc.

Some objection could be offered to the location of the kitchen and dining room in the basement story, in view of the fact that some of the patients would not be able to go up and down stairs to their meals; but the infirmary will be large enough to accommodate most of the infirm patients. Arranging the kitchen and dining room in the basement removes them with all their objectionable features from the floor where the patients will spend the time they are indoors during the day. It is the object to have the patients occupy the first floor exclusively during the day, as most of the sleeping apartments have been arranged on the second floor. This, I think, insures a better sanitary condition of the buildings throughout, and will enable us to keep the sleeping apartments in a well ventilated, orderly condition during the day.

The plan provides for but comparatively few single or isolated rooms; and this I think advisable, in order to give the night attendants a better opportunity to be in easy reach of all the patients, so that during seizures, accidents can largely be prevented. With a number of single rooms, it seems to me that many accidents might occur by patients falling, or turning on their faces and smothering in the bed clothing. I am aware that many objections have been offered to large dormitories, but when they are arranged to accommodate not more than eight or ten patients, as we have them planned, I am of the opinion that they are most desirable for the reasons above mentioned.

It is the intention to care for the insane epileptics, as well as for those who have not been declared insane, and, while I have advised against this at all times, still I have come to the conclusion that perhaps it is well to do this. We all fully understand that even with the lightest forms of epilepsy the mentality and proper conduct of the epileptic is uncertain, and that at all times he is wholly irresponsible for his acts; that epilepsy in all forms tends to the ultimate destruction of the mind; and that the insane epileptics differ only in degree from those who have not been considered insane. In the early history of insane epileptics, the mental disturbance is transient, and the length of time a normal condition is maintained depends upon the proper control of their diet and habits and upon favorable environment. This history applies with variations to every case until the stage of dementia is reached, when the patient becomes untidy in his habits and disorderly at all times. This being true, and with an institution having grounds large enough for separate and distinct places for recreation and exercise, and cottages sufficiently separated to prevent one class from disturbing another, I cannot see the objection to caring for both classes under one management.

I believe it would be a great injustice to place epileptics suffering from temporary mental excitement in hospitals for the insane, where they would be deprived of conditions and skilled medical treatment and nursing that would be most conducive to their improvement, if it were at all possible to place them in an institution especially designed and equipped for the care and treatment of this class. It is, perhaps, a greater injustice to subject the patients in an insane hospital to association with epileptics whose habits and conduct are so different.

It is our purpose to keep the classes distinctly separated, by setting apart cottages for those who are suffering from temporary mental excitement or disturbance, and other cottages for those who are permanently deranged or demented. It has occurred to me that after an epileptic has been declared insane and confined in an insane asylum there might be some difficulty in getting the management of an institution for epileptics to admit the case, even after the patient has become quiet, and the expense of transfer, where the distance is so great between the institutions, as it is in our State, would be considerable.

In conclusion, I think I can refer with pardonable pride to the liberality of the people of Texas in always being ready to make ample provision for the proper care of the unfortunate and defective people found within her borders. Within a few years I confidently expect to see the colony now under course of construction the equal in equipment, if not in management, of any similar institution in this or any other country.

## Figures and Tables

**Figure f1:**